# Transgenic *Anopheles gambiae* Expressing an Antimalarial Peptide Suffer No Significant Fitness Cost

**DOI:** 10.1371/journal.pone.0088625

**Published:** 2014-02-06

**Authors:** Clare C. McArthur, Janet M. Meredith, Paul Eggleston

**Affiliations:** Centre for Applied Entomology and Parasitology, Keele University, Keele, Staffordshire, United Kingdom; Centro de Pesquisas René Rachou, Brazil

## Abstract

Mosquito-borne diseases present some of the greatest health challenges faced by the world today. In many cases, existing control measures are compromised by insecticide resistance, pathogen tolerance to drugs and the lack of effective vaccines. In light of these difficulties, new genetic tools for disease control programmes, based on the deployment of genetically modified mosquitoes, are seen as having great promise. Transgenic strains may be used to control disease transmission either by suppressing vector populations or by replacing susceptible with refractory genotypes. In practice, the fitness of the transgenic strain relative to natural mosquitoes will be a critical determinant of success. We previously described a transgenic strain of *Anopheles gambiae* expressing the Vida3 peptide into the female midgut following a blood-meal, which exhibited significant protection against malaria parasites. Here, we investigated the fitness of this strain relative to non-transgenic controls through comparisons of various life history traits. Experiments were designed, as far as possible, to equalize genetic backgrounds and heterogeneity such that fitness comparisons focussed on the presence and expression of the transgene cassette. We also employed reciprocal crosses to identify any fitness disturbance associated with inheritance of the transgene from either the male or female parent. We found no evidence that the presence or expression of the effector transgene or associated fluorescence markers caused any significant fitness cost in relation to larval mortality, pupal sex ratio, fecundity, hatch rate or longevity of blood-fed females. In fact, fecundity was increased in transgenic strains. We did, however, observe some fitness disturbances associated with the route of inheritance of the transgene. Maternal inheritance delayed male pupation whilst paternal inheritance increased adult longevity for both males and unfed females. Overall, in comparison to controls, there was no evidence of significant fitness costs associated with the presence or expression of transgenes in this strain.

## Introduction

Mosquito species are important global vectors of diseases such as malaria, dengue, yellow fever, filariasis and encephalitis. Of these, malaria is arguably the greatest problem, with up to 40% of the human population at risk of infection and around one million deaths each year. One of the major routes to disease control is to tackle the mosquito vectors themselves, primarily with insecticides. However, as a result of these interventions, mosquitoes are becoming increasingly resistant to pesticides [1] and additional approaches are clearly required. The feasibility of utilising transgenic mosquito technology to control disease transmission has been demonstrated [2]–[11]. However, as with any genetic intervention, the ultimate goal of disease control through transgenic technology will rely upon effective inheritance of the transgene and its introgression into field populations. For such technology to become a viable part of an integrated pest management programme, genetically modified mosquitoes must be able to compete successfully for available resources. This requires either the absence of a fitness load associated with the genetic modification, or else a modest fitness load that may require the use of a gene drive mechanism to help fix the transgene in the population [12], [13]. To achieve this outcome it is important that transgenic mosquitoes, destined for mass rearing and field release, have minimal fitness costs associated with transformation and transgene expression.

The overall fitness of a given transgenic strain will depend upon a number of factors that each require investigation prior to consideration for use in field trials. First, the introduction of a designed intervention against the pathogen may have unintended fitness consequences for transgenic strains. For example, the introduction or over-expression of effector genes may have a negative impact on fitness [14] and the expression of different effector genes (even under control of the same regulatory sequences) can have differing fitness costs in transgenic strains [15]. Secondly, factors that are independent of the effector gene or refractoriness to disease transmission may impact fitness. For example, fluorescence marker expression cassettes are typically employed to allow identification of transformed individuals [16]. There are reports of adverse fitness effects on transgenic mosquitoes caused by simple promoter-marker combinations [17], [18]. This may depend upon the regulatory sequences chosen for fluorophore expression but is most likely a result of insertional mutagenesis following transgene insertion. Insect transgenesis is typically mediated by transposons that have an essentially random pattern of genome integration [19]. There is therefore an associated potential to disrupt native gene function [14]. This can impose fitness costs, but is largely dependent on the choice of transposon. For example, the *P* element exhibits a strong tendency to integrate into gene promoter regions whereas the *piggyBac* element appears to have evolved to largely avoid host gene disruption [20]. In addition, the essentially random nature of transposon integration can result in position effects whereby transgene(s) are influenced by the proximity of other gene regulatory sequences, or epigenetic modifications. In our experience, position effects can have a significant impact on the observed levels of both fluorescence marker and effector gene expression [9], [16], [21]. It follows that transgene location is an important factor to consider and that fitness implications associated with transgene expression could be either ameliorated or compounded by genomic location. Thirdly, and perhaps most importantly, inbreeding and loss of genetic heterogeneity as a result of the genetic bottlenecks inherent in genetic modification may impact on fitness. Transgenic strains are established from very few founder individuals (typically from a single transgenic adult) and are then maintained as small laboratory populations. As a result, the resulting strains may carry fixed deleterious alleles with the potential to impact on fitness [18]. Indeed, the detrimental effects of inbreeding on mosquitoes in the absence of transgenes has been previously demonstrated [13].

A range of fitness studies have been undertaken to determine the impact of transgenesis on mosquito biology in both *Anopheles stephensi* [6], [10], [12], [15], [18], [22]–[24] and *Aedes aegypti* [13], [25], [26]. Significant fitness disturbances have been reported for some transgenic strains but the effect is not uniform across similar strains and can be impacted by any or all of the factors discussed above. The effector gene itself has been directly responsible for an impact on fitness in two studies [6], [15]. Corby-Harris *et al.* reduced malaria parasite infection by increasing *Akt* signalling in the mosquito midgut [6]. Lifespan was also reduced in these transgenics, even in non-blood-fed females, which was attributed to leaky expression from the carboxypeptidase promoter. A reduction in fitness was also observed when phospholipase A2 from honeybee venom was expressed from the carboxypeptidase promoter [15]. This was presumed to be as a consequence of internal damage to the midgut and was resolved by expression of a mutant protein [7]. The same researchers observed no fitness disturbance in hemizygous transgenic mosquitoes expressing an alternative antimalarial effector gene, SM1, in the midgut [15]. A fitness advantage compared to wild-type was even reported when SM1 transgenic mosquitoes were maintained on *Plasmodium* infected blood [22]. Isaacs *et al*. [10] also reported no fitness load associated with the expression of single-chain antibodies when compared to the control strain. Since females of one strain exhibited an increased median lifespan, there may once again be an associated fitness advantage. Where a fitness load has been identified, it has more often been attributed to position effects or insertional mutagenesis, compounded by inbreeding, rather than expression of the transgene itself. Such a fitness load was reported for three independent, homozygous transgenic strains expressing SM1 into the haemocoel [12]. Due to the particular inbreeding issues associated with generating homozygous transgenic strains, which are likely to exacerbate founder effects and result in inbreeding depression, it is not surprising that fitness loads have also been identified in other homozygous strains [18], [25], [26]. Such fitness disturbance is more pronounced when assessing changes in transgenic allele frequencies over time rather than measuring fitness parameters directly [12]. Fitness loads associated with position effects are dependent upon transgene location and result in a lack of uniformity between strains such as those reported for fitness profiles of four independent hemizygous *An. stephensi* docking strains [23]. However, this study concluded that none of the strains carried significant fitness burdens compared to their non-transgenic counterparts. Other studies, designed to separate the effect of inbreeding from that of genetic modification have concluded that transgenesis has an effect on fitness that is additional to that of inbreeding. Koenraadt *et al*. [13] found that transgenic *Ae. aegypti* fared least well in comparison to both wild-type and an inbred strain for most fitness parameters measured, but that the inbred strain was also less fit than wild-type. Another study in which transgenic *Ae. aegypti* were outcrossed to the unmodified strain for five generations in order to minimise inbreeding effects, observed significant differences in all measured fitness parameters when comparing the outcrossed transgenic strain to wild-type [13]. It was therefore suggested that in this instance transgene products may be responsible for the overall reduction in fitness of the transgenic strain compared to wild-type.

It is clear from these various fitness studies that, in designing comparisons between transgenic and non-transgenic strains, care needs to be taken to equalize as far as possible the genetic backgrounds and heterogeneity of the strains under comparison. Only then can any inference be drawn about the possible impact of the genetic modification itself. The present study was designed to investigate fundamental fitness parameters of the transgenic *An. gambiae* EVida3 strain [9]. This strain expresses a synthetic antimicrobial peptide, Vida3 [27], into the female midgut lumen following a blood-meal. EVida3 was generated using the phiC31 site-specific integration system [28], whereby the effector gene cassette was integrated into a previously characterised docking site on chromosome 3R. Therefore, in addition to the Vida3 cassette, the strain expresses two fluorescence markers (ECFP and DsRed2), both under control of the eye-specific 3×P3 promoter. The fitness costs associated with inbreeding have been well documented [12], [13] and a study modelling the population dynamic effect following the introduction of a genetically modified mosquito into a wild-type population concluded that the relative fitness of the resulting hemizygotes is crucial for a successful release [29]. Hemizygotes were therefore chosen for this study and were generated by outcrossing EVida3 homozygotes to the parental KIL laboratory strain from which they were derived in order to equalize genetic backgrounds [25]. Hemizygotes were established using reciprocal crosses between EVida3 and KIL to enable comparisons between strains that inherited the transgene via the maternal parent (EV3M) or paternal parent (EV3P). This assessment of fitness parameters is an important step towards increasing our understanding of the biology of transgenic strains such as EVida3 and their potential performance in the field.

## Results

### Larval Mortality

**Figure 1:**
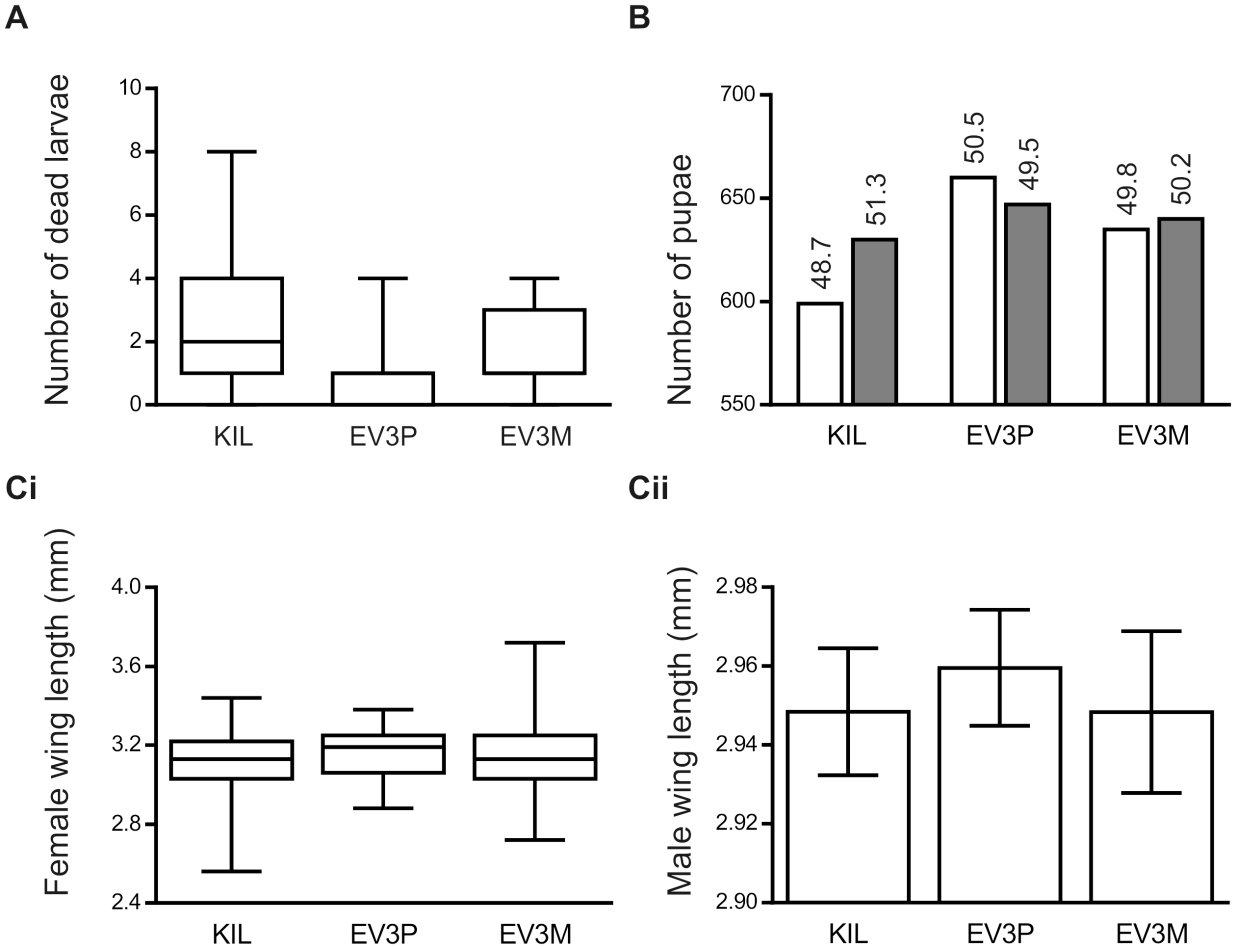
Larval mortality, pupal sex ratios and wing lengths. Data represent comparisons between the control (KIL) and hemizygous transgenic strains (EV3P and EV3M). (A) Larval mortality data are pooled over 3 independent experiments, each with 15 replicates of 30 individuals per strain. Boxplots show the interquartile range (open boxes), the median (horizontal line for KIL but coinciding with the interquartile range at 1 for both EV3P and EV3M) and the maximum and minimum values (vertical lines). There were no significant differences between strains (*p* = 0.368). (B) Numbers of pupae and pupal sex ratio data are pooled over 3 independent experiments, each with 15 replicates of 30 individuals per strain. The percentage of females (open bars) and males (shaded bars) for each strain is indicated. Statistical analysis showed no significant difference in sex ratios between strains (*p* = 0.673). (C) Wing length data are pooled over 3 independent experiments, each involving 100 female and 100 male mosquitoes for each strain. (Ci) Female wing lengths were non-normally distributed and are represented as boxplots showing the median (horizontal line), interquartile range (boxed) and maximum and minimum values (vertical lines). There were no significant differences between strains (*p* = 0.112). (Cii) Male wing lengths followed a normal distribution and are represented as open bars with standard errors (vertical lines). There were no significant differences between strains (*p* = 0.590).

Control (KIL) and hemizygous transgenic (EV3P and EV3M) larvae were generated and reared following detailed protocols to minimise the impact of environmental variance. Three independent experiments were established, each comprising 15 replicates with 30 individual mosquitoes per strain. Replicates were individually randomised within the insectary to minimise confounding placement effects. Larval death was recorded at the same time each day until all larvae had either pupated or died. Mean larval mortalities were 2.69 (9.0%), 0.96 (3.2%) and 1.67 (5.6%) for KIL, EV3P and EV3M respectively. Since these data were non-normally distributed, the medians with associated ranges were plotted (Figure 1A). Statistical analysis using a non-parametric Kruskal-Wallis test revealed no significant difference in larval mortality between strains (*p* = 0.368).

### Pupal Sex Ratio

The number and sex of all pupae recovered from the larval mortality experiment described above were analysed using data pooled across all replicates from 3 independent experiments. From a total of 1350 larvae of each strain, 1229 (91%), 1307 (96.8%) and 1275 (94.4%) larvae survived to pupation for KIL, EV3P and EV3M, respectively. The sex ratios (female:male) were 48.7∶51.3 (KIL), 50.5∶49.5 (EV3P) and 49.8∶50.2 (EV3M), respectively (Figure 1B). These data were analysed using a Chi-squared contingency test which revealed no significant difference in sex ratio between the three strains (χ^2^ = 0.792; 2 degrees of freedom; *p* = 0.673).

### Wing Length

Mosquitoes for wing length measurements were collected from the longevity study described below. Wing lengths were measured for both males and females from control (KIL) and hemizygous transgenic (EV3P and EV3M) strains. Data were pooled across 3 independent experiments each comprising 100 males and 100 females of each strain. Average female wing lengths for each strain were 3.11 mm (KIL), 3.16 mm (EV3P) and 3.16 mm (EV3M) respectively (Figure 1Ci). Female wing length data were non-normally distributed (D’Agostino-Pearson omnibus test) and were compared using the Kruskal-Wallis test, which revealed no significant difference between strains (*p* = 0.112). Average male wing lengths for each strain were 2.95 mm (KIL), 2.96 mm (EV3P) and 2.95 mm (EV3M) respectively (Figure 1Cii). Male wing lengths followed a normal distribution (D’Agostino-Pearson omnibus test) and were analysed using ANOVA which revealed no significant difference between strains (*p* = 0.590).

### Age at Pupation

**Figure 2:**
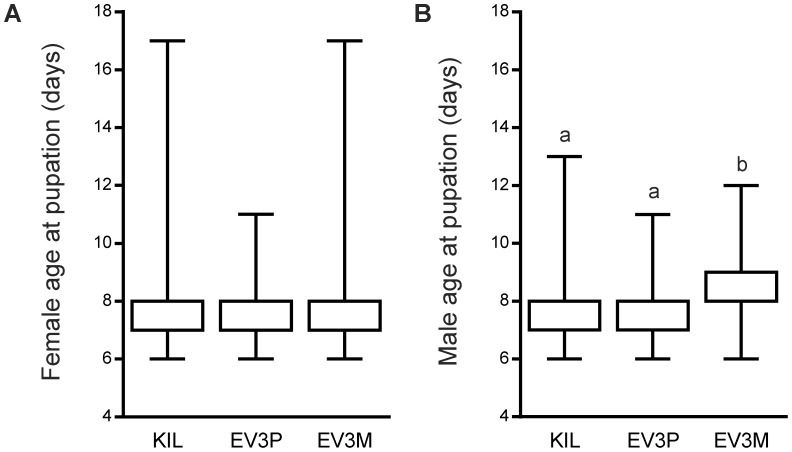
Age at pupation. Data represent comparisons between the control (KIL) and hemizygous transgenic strains (EV3P and EV3M) and show age at pupation for females (A) and males (B). Data are pooled over 3 independent experiments, each with 15 replicates of 30 individuals per strain. Boxplots show the interquartile range (open boxes) with maximum and minimum values (vertical lines). Different lower case letters (a, b) identify significant differences. The median pupation age is 8 days for all data sets. The age at pupation for females was not significantly different between strains (*p* = 0.367) but there was a significant difference in male pupation rates between strains (*p*<0.0001). Further analysis identified a significant difference between EV3M and KIL (*p*<0.0001) and between EV3M and EV3P (*p*<0.0001).

Data for age at pupation for control (KIL) and hemizygous transgenic (EV3P and EV3M) strains were collected from the larval mortality experiment described above. Age at pupation was recorded separately for males and females of each mosquito strain. For females, the mean age at pupation was 7.76 days (KIL), 7.72 days (EV3P) and 7.76 days (EV3M) respectively. Statistical analysis of pooled data using the non-parametric Kruskal-Wallis test revealed no significant difference in age at pupation between strains (*p* = 0.367; Figure 2A). For males, the mean age at pupation was 7.66 days (KIL), 7.59 days (EV3P) and 8.32 days (EV3M) respectively. Statistical analysis of pooled data (Kruskal-Wallis test) revealed a significant difference in age at pupation between strains (*p*<0.0001; Figure 2B). Further analysis using Dunn’s multiple post-test for pair-wise comparisons identified a significant difference between the age at pupation of EV3M males when compared to either KIL or EV3P (*p*<0.0001 by Mann Whitney test in both cases; Figure 2B). There was no significant difference in age at pupation between KIL and EV3P males (Figure 2B).

### Adult Longevity

**Figure 3:**
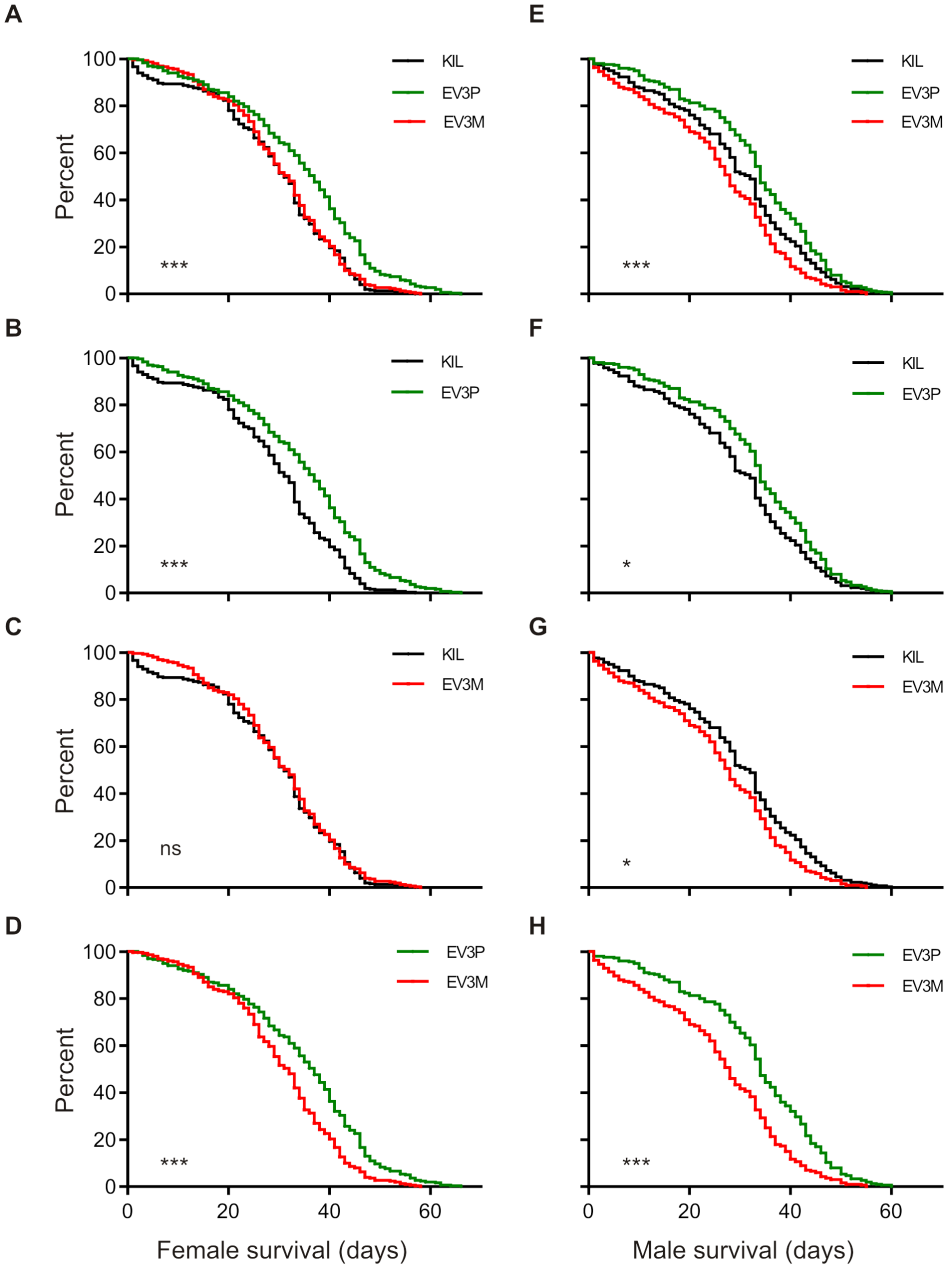
Adult longevity survival curves. Graphs represent comparisons between the control (KIL, black) and hemizygous transgenic strains (EV3P, green and EV3M, red) and show Kaplan-Meier adult survival curves for females (A–D) and males (E–H). (A) shows survival curves for females of all three strains. (B–D) show paired comparisons between strains for female survival. (E) shows survival curves for males of all three strains. (F–H) show paired comparisons between strains for male survival. All survival curves represent pooled data from three independent experiments, each with 10 replicates of 10 adults for each strain. Significance levels are displayed on each graph (***, *p*<0.0001; *, *p*<0.05; ns; not significant).

Mosquitoes were reared following detailed protocols to minimise environmental variance and adult longevity was recorded separately for males and females of each strain. Data were pooled from 3 independent experiments, each comprising 100 male and 100 female mosquitoes per strain, separated into 10 replicates of 10 newly eclosed mosquitoes. Adults were reared on sugar and water and dead mosquitoes were removed and recorded daily until all mosquitoes had died. For females, average adult longevity was 28.57 days (KIL), 34.32 days (EV3P) and 30.37 days (EV3M) respectively. For males, average adult longevity was 29.50 days (KIL), 33.16 days (EV3P) and 26.24 days (EV3M) respectively. Pooled data were analysed for statistical significance using the log-rank (Mantel-Cox) test. For both KIL and EV3P, adult longevity did not differ significantly between males and females (*p* = 0.1649 and *p* = 0.0585 respectively; compare Figures 3A, 3E) whereas for EV3M, females were significantly longer lived than males (*p* = 0.0023; compare Figures 3A, 3E). Further analysis revealed a significant difference in longevity between strains for both females (Figure 3A; *p*<0.0001) and males (Figure 3E; *p*<0.0001). Pooled female and male data were then separately subjected to pair-wise comparisons to determine where the major differences in longevity lay. For females, there was no significant difference in longevity between KIL and EV3M (Figure 3C; *p* = 0.462) but significant differences were observed between KIL and EV3P (Figure 3B; *p*<0.0001) and between EV3P and EV3M (Figure 3D; *p*<0.0001). For males, significant differences were identified in all pair-wise comparisons, between KIL and EV3P (Figure 3F; *p* = 0.004), between KIL and EV3M (Figure 3G; *p* = 0.001) and between EV3P and EV3M (Figure 3H; *p*<0.0001).

### Fecundity and Hatch Rate

**Figure 4:**
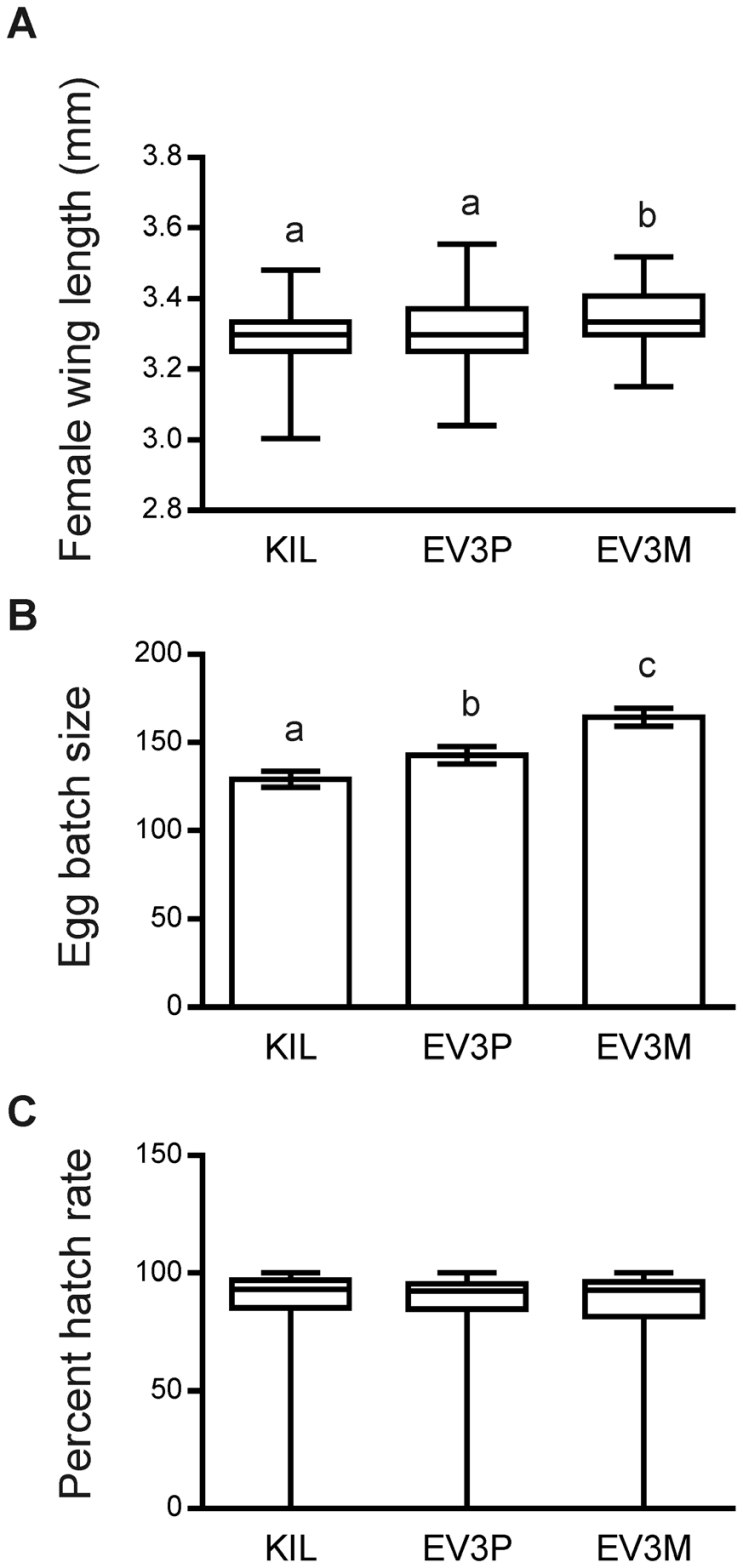
Fecundity and fertility. Data represent comparisons between the control (KIL) and hemizygous transgenic strains (EV3P and EV3M). All data are pooled from 3 independent experiments. Different lower case letters (a, b, c) identify significant differences. (A) Wing length of adult females, reared for fecundity and longevity experiments with blood-meals. Boxplots show the interquartile range (open boxes), the median (horizontal line) and the maximum and minimum values (vertical lines). EV3M wing length is significantly longer than both KIL (*p* = 0.0003) and EV3P (*p* = 0.0106). (B) Egg batch sizes, collected from single females showing the mean (open bars) and standard errors (vertical lines). The control KIL females produced significantly smaller egg batches than the transgenic strains EV3P (*p* = 0.0411) and EV3M (*p*<0.0001). EV3M egg batches were also significantly larger than EV3P (*p = *0.0025). (C) Percent hatch rate from egg batches collected in (B). Boxplots show the interquartile range (open boxes), the median (horizontal line) and the maximum and minimum values (vertical lines). There are no significant differences in hatch rate between the control and either transgenic strain (*p* = 0.773).

Mosquitoes for fecundity and hatch rate assessments were reared together with those for the blood-feeding longevity experiments detailed below, following detailed protocols to minimise environmental variance. Representative samples from each of 3 independent experiments were measured for wing length. Average female wing lengths for each strain were 3.29 mm (KIL), 3.30 mm (EV3P) and 3.34 mm (EV3M) respectively (Figure 4A), which were larger than those reared previously (Figure 1Ci). These non-normally distributed data (D’Agostino-Pearson omnibus test) identified a significant difference in wing length (*p* = 0.0013) using the Kruskal-Wallis test for this set of samples. Pair-wise analysis (Mann-Whitney test) showed that EV3M females were significantly larger than both EV3P (*p* = 0.011) and KIL (*p* = 0.0003), but that there was no significant difference in size between EV3P and KIL (*p* = 0.507). Egg batch sizes collected from individual females followed a normal distribution (D’Agostino-Pearson omnibus test) and were analysed using ANOVA, which also revealed significant differences between strains (*p*<0.0001; Figure 4B). Mean egg batch sizes were 129.1 (KIL), 142.7 (EV3P) and 164.2 (EV3M) respectively and pair-wise analysis (unpaired t-test with Welch’s correction) identified significant differences between all 3 strains. EV3M egg batches were significantly larger than both EV3P (*p* = 0.0025) and KIL (*p*<0.0001), which is most likely attributable to the larger wing length of EV3M females (Figure 4A). A significant difference was also identified between KIL and EV3P egg batches (*p* = 0.0411), where EV3P produced slightly larger egg batches than the control, although their wing lengths were not significantly different (Figure 4A). The mean hatch rates for egg batches collected above were 85.72% (KIL), 86.79% (EV3P) and 86.36% (EV3M; Figure 4C). Statistical analysis of pooled data (Kruskal-Wallis test) revealed no significant differences in hatch rates between strains (*p* = 0.773).

### Female Longevity Following Blood-feeding

**Figure 5:**
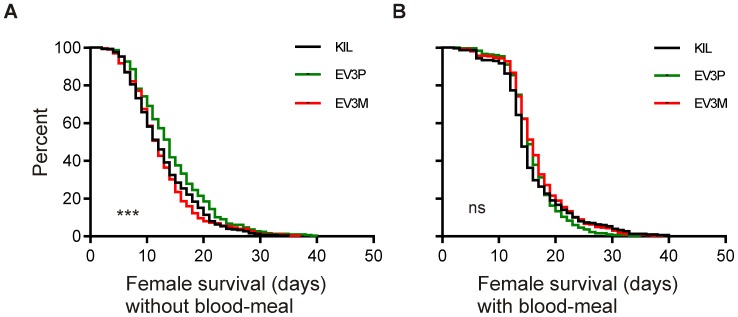
Adult longevity survival curves following mating and blood-feeding. Graphs represent comparisons between the control KIL (black) and hemizygous transgenic strains EV3P (green) and EV3M (red) and show Kaplan-Meier adult survival curves established with 3 day-old mated females. All survival curves represent pooled data from three independent experiments, each with 4 replicates of 25 adults for each strain. (A) Female survival in the absence of blood-feeding differs significantly between strains (*p* = 0.0004), with EV3P surviving significantly longer than either KIL (*p* = 0.002) or EV3M (*p* = 0.0002). (B) Female survival following blood-meals at days 0 and 5 of the experiment does not differ significantly between strains (*p* = 0.154). Significance levels are displayed on each graph (***, *p*<0.0001; *, ns; not significant).

Control and hemizygous transgenic mosquitoes were reared as detailed in Materials and Methods and adults collected daily. Three days post-eclosion, female sub-populations were either left unfed or offered a blood-meal (designed to stimulate expression of the Vida3 peptide from the carboxypeptidase promoter), from which fully engorged females were selected. Blood-fed cages were offered oviposition sites two and three days post-blood-meal and a second blood-meal eight days post-eclosion. Dead mosquitoes were removed and recorded daily and data were pooled from 3 independent experiments, each comprising 4×25 non-fed and 4×25 blood-fed mosquitoes per strain. For mated but non-blood-fed females average longevity was 12.90 days (KIL), 14.47 days (EV3P) and 12.55 days (EV3M), which is considerably shorter than for females reared previously (Figure 3), which were neither mated nor subjected to periods of sugar starvation. Data were analysed for statistical significance using the log-rank (Mantel-Cox) test which revealed a significant difference between strains (*p* = 0.0004; Figure 5A). Separate pair-wise comparisons identified unfed EV3P females to be significantly longer lived than both KIL (*p* = 0.002) and EV3M (*p* = 0.0002), although there was no significant difference in longevity between unfed KIL and EV3M (*p* = 0.524). For females that were blood-fed, more than 95% of surviving individuals from all three strains took a second blood-meal five days later. Average longevity for blood-fed females was 15.81 days (KIL), 16.07 days (EV3P) and 16.68 days (EV3M). Analysis by log-rank (Mantel-Cox) revealed no significant difference between blood-fed females (*p* = 0.154; Figure 5B). Comparing Figures 5A and 5B, there was a significant increase in longevity in sugar-starved females that were blood-fed compared to non-fed for both KIL and EV3M (*p*<0.0001 for both) but not EV3P (*p* = 0.159).

## Discussion

This study compared the fitness of hemizygous transgenic mosquitoes expressing an antimalarial effector gene [9] to the wild-type host strain from which they were derived. The hemizygous strains were established from reciprocal crosses between KIL and EVida3 homozygotes such that the transgene was inherited from either the paternal (EV3P) or the maternal (EV3M) parent. Hemizygotes were used in an attempt to equalize the genetic backgrounds and heterogeneity of the strains under comparison and to minimise any deleterious impacts of inbreeding during creation of the transgenic strains. Our results revealed no significant difference between transgenic and wild-type mosquitoes for four of the fitness parameters measured, namely larval mortality, pupal sex ratio, hatch rate and longevity of blood-fed females. Some differences were identified for age at pupation, body size (wing length), fecundity and longevity of sugar-fed adults. However, these differences did not suggest transgenic inferiority.

Whilst larval mortality did not differ between strains, the overall mean mortality of around 6% was significantly lower than that reported for *An. gambiae* in natural situations, where rates can be as high as 92–98%. This can be due to varying factors such as the presence or absence of parasites, pathogens and predators within larval pools, inter- and intra-specific competition and abiotic factors such as temperature and rainfall [30], [31]. With the exception of intra-specific competition, none of these macro-environmental variables are present in the laboratory setting. However, it has been reported that higher larval mortality rates from intra-specific or intra-strain competition may result in larger, more fecund adult females, resulting in no net fitness disadvantage [13].

No distortion in pupal sex ratios was found between strains since there were no significant departures from the expected 1∶1 ratio of males to females. This is consistent with the facts that (a) the transgene is autosomal, (b) effector gene expression is predominantly seen in blood-fed adult females and (c) fluorescence markers are regulated by the 3×P3 promoter which is equally expressed in both sexes. It has been reported that carboxypeptidase mRNA is detectable in the pupal gut [32]. We therefore infer that mRNA and translated peptides from effector genes such as Vida3, which are expressed from the carboxypeptidase propter, are presumably also detectable in pupal tissues. However, our data show clearly that expression of the Vida3 effector peptide has no influence on pupal sex ratio.

Age at pupation was remarkably consistent, with no significant differences between females of any strain and none between males of the KIL and EV3P strains. However, EV3M males were significantly slower to pupate than either KIL or EV3P males. Mating in *An. gambiae* occurs in male swarms, which await and then compete for, arriving females. Such swarms apparently lack a female-choice component, placing more emphasis on male competition [33]. It has also been suggested that slower development of transgenic mosquito larvae can delay the onset of male sexual maturity [12]. Thus, if EV3M males take longer to reach pupation and sexual maturity than their wild-type counterparts, this could indicate a fitness disadvantage that might impact on transgene transfer to subsequent generations under field conditions. Conversely, EV3P males pupated at the same age and reached eclosion within a similar timeframe to KIL.

There were no significant differences in wing length between strains for either males or females reared for the initial experiments. However, EV3M females subsequently reared for the assessment of fecundity, fertility and longevity following blood-feeding had significantly longer wing lengths. Wing length has been documented as a good indicator of body size for anopheline mosquitoes [34]. Furthermore, body size provides a good indicator of the blood-meal volume taken by females, which is a primary determinant of fecundity [35], [36]. In one field study, smaller female mosquitoes, hand caught in Tanzania, were more often pre-gravid than their larger counterparts, requiring two or three blood-meals before completing the first gonotrophic cycle [35]. This study defined smaller females as having wing lengths of less than 3 mm whereas the laboratory strain females we describe here all had wing lengths in excess of 3 mm, indicative of optimized rearing conditions. Other studies of adult body size have reported that male *An. gambiae* preferentially select larger females, even when large numbers of males were competing for mates, thus suggesting that larger females are preferred due to their increased egg-carrying capacity [37]. Body size has also been reported to impact on male competitiveness during sexual selection in mosquito swarms [38]. This study suggested that the most successful males were of intermediate size (six times more likely to achieve copulation during one swarming event) although, within size groups, the mated males were larger than those which did not mate. It is therefore clear that body size of both males and females is an important determinant of reproductive success in *An. gambiae*. We therefore concentrate on the comparisons between EV3P and KIL females in the discussion of experiments involving blood-feeding below, where there were no significant differences in wing length. Since the use of transgenic mosquitoes in a disease control program would involve the release of homozygous males, it is important that such males are not compromised in body size and we have found no such evidence in studies of EVida3 homozygotes (unpublished data).

Egg batches were significantly larger in transgenic line EV3M, which is entirely consistent with the longer wing length observed for these experiments. However EV3P females also produced larger egg batches than the wild-type control. Since there was no significant difference in wing length between EV3P and KIL females, we conclude that this transgenic strain is actually more fecund than the control. Regardless of egg batch sizes, hatch rates were not significantly different between strains. Primigravida EV3P females could therefore be expected to produce more offspring than their KIL counterparts.

Longevity experiments on mated females that went through two gonotrophic cycles were designed to determine whether upregulation of the transgene following naïve blood-meals would impose a fitness cost. The *An. gambiae* carboxypeptidase promoter has previously been used to drive blood-meal inducible transgene expression in several *An. stephensi* transgenic lines. A fitness load was reported in two of these following naïve blood-meals, both directly linked to the action of the transgenes themselves [6], [15]. For two different transgenic lines no significant effect on lifespan was observed following midgut transgene expression [15], [24]. We observed no significant difference in longevity between transgenic and non-transgenic strains offered blood-meals. Thus, for EV3P females, where no significant differences to KIL were observed for wing length, hatch rate of progeny or lifespan, but for which egg batch sizes were significantly larger, there is the possibility of producing more progeny in a lifetime. We conclude that in the hemizygous transgenic mosquitoes tested here, neither the presence or the expression of the effector gene or fluorescence markers have a significant impact on larval mortality, pupal numbers and sex ratios, egg hatch rate or longevity following blood-feeding, and may in fact offer a fitness advantage since fecundity was increased.

The greatest differences between strains were observed when assessing the longevity of non-mated and mated, non-fed adults. In the non-mated experiment, there was no significant difference between KIL and EV3M females but KIL males were significantly longer lived than EV3M males. Conversely, both EV3P males and females were significantly longer lived than their KIL counterparts. This increase in lifespan, whereby EV3P females were significantly longer lived than KIL, was also observed for females that were mated and sugar starved. Thus, we identified significant differences between strains for adult longevity but, perhaps unexpectedly, the wild-type was not consistently fittest. This suggests that there can be no *a priori* assumption of transgenic inferiority. We also observed some significant differences in longevity between reciprocal transgenic strains, whereby EV3P tended to be longer lived than their EV3M counterparts. This was unexpected since the two strains differ only in the route of inheritance of the transgene, from either the paternal or maternal parent. A similar situation was also evident from the data on age at pupation where male EV3M pupation was significantly slower than that of EV3P males. We believe this to be the first report of fitness disturbances associated with the route of inheritance of a transgene, although the mechanism remains unclear. It seems unlikely that such differences are a function of inbreeding, since both transgenic strains would have identical inbreeding coefficients. We can therefore speculate that the route of inheritance of the transgene, rather than the transgene *per se*, can have pleiotropic effects on genes that influence fitness traits. This could arise from interactions with genetic determinants such as mRNA or proteins derived from the oocyte, or perhaps as a result of epigenetic mechanisms such as parental imprinting. It does, however, mean that the route of transgene inheritance must be considered in any biological or genetic characterization of transgenic strains destined for field release.

The consequences of longevity variations in the field are likely to be complex. A longer lived female may undergo more gonotrophic cycles and thus increase reproductive fitness. Conversely, longevity may be less important to males, who can mate rapidly with multiple females even if lifespan is curtailed. There may even be a competitive advantage for those males that can mate early in life, although there is evidence to suggest a limit on mating capacity due to resource costs [39]. However, longer lived males will have greater opportunity to replenish resources and continue mating. If males are compromised in terms of reproductive fitness, perhaps because they take longer to pupate, and are less competitive early in life, then a reduced longevity would further compromise fitness. In addition, long-term survival in males is associated with high lipid content and larger body size [40]. However, a lower lipid content observed in laboratory-bred males (compared to natural males) could indicate a reduced reproductive potential [41]. Ng’habi *et al*. [38] also showed that larger mosquitoes were consistently longer-lived than intermediate sized males. They suggested that, although longer lived males have more opportunity to mate over their lifetime, this may not offset the fitness disadvantage their size confers since intermediate-sized males were found to be six times more likely to achieve copulation.

The laboratory based fitness studies carried out here can not be truly representative of a field situation where mosquito larval stages will encounter increased resource competition, adults will forage for food (sugar and blood-meals) and both will be subjected to additional stress factors such as predation and pathogens. In all but our final longevity study (Figure 5B), females did not receive a blood-meal and so did not upregulate expression of the Vida3 peptide or enter a gonotrophic cycle, all of which may affect longevity. Taking a blood-meal in the field risks exposure to pathogens, such as *Plasmodium* spp., which can upregulate immune responses, such as melanisation, and possibly decrease life expectancy [42]. The melanisation response is rarely seen in co-evolved *Plasmodium*-anopheline combinations [43] and may therefore lead to a greater difference than expected between transgenic strains such as EVida3 and wild-type competitors. For transgenic refractory females, a reduction in average longevity due to upregulated immune responses [42] could be offset by reduced efficiency as a disease vector, offering an overall benefit. However, transgenic mosquitoes not previously exposed to pathogens or parasites in the laboratory, may elicit a greater immune response compared to native wild-type mosquitoes. Interestingly, transgenic malaria refractory lines were reported to be longer lived and more fecund when fed routinely on *P. berghei*-infected blood in the laboratory [22]. We also noted that the virgin males used in our longevity studies lived longer than expected from general observation of stock populations within the same laboratory. This is consistent with the reports of Dao *et al*. [39] who showed that *An. gambiae* males exposed to females for six days had reduced longevity compared to virgin males. Fitness variations resulting from mating behaviour are likely to be even more pronounced in field situations where swarming is presumably more vigorous than in confined insectary conditions.

Overall, the fitness parameters investigated in this study showed no evidence that the presence or expression of the Vida3 transgene or the associated fluorescence markers had a negative impact on fitness. Where significant differences between wild-type and transgenic strains were identified, they were not consistent across reciprocal hemizygous strains or between sexes. In addition, for some analyses, the transgenic lines outperformed their wild-type counterparts. Moreover, we identified variations in relative fitness associated with the route of transmission of the transgene (either from the male or female parent) that may need to be considered in the design and deployment of transgenic mosquitoes for field release. Such laboratory assessments of fitness are clearly necessary for initial characterization of newly generated transgenic strains. On their own, however, they would not be sufficient and would need to be supported by semi-field or field assessments of fitness prior to any consideration for use in releases aimed at disease control.

## Materials and Methods

### Generation of Test Strain Mosquitoes

Mosquitoes were maintained at 26°C ±1°C and 80% RH in a 12-hour light: 12-hour dark photoperiod. Trays (35 cm×25 cm) of wild-type KIL and homozygous transgenic EVida3 larvae (L_1_ stage) were set up with equal numbers and reared following a standardised protocol until pupation. Pupae from both lines were sexed and equal numbers of the resulting adults put into each of three crosses to produce one KIL control population and two reciprocal hemizygous EVida3 strains (EV3P: EVida3 males with KIL females and EV3M: EVida3 females with KIL males). This protocol for generating experimental mosquitoes ensured the resulting wild-type and hemizygous strain individuals would be comparable. Adults were maintained on water and 10% glucose *ad libitum*. 4–7 day old adult mosquitoes were sugar starved for 18–24 hours and females blood-fed on defribrinated horse blood (TCS Biosciences Ltd, Buckingham, UK) using a Hemotek membrane feeding system (Discovery Workshops, Accrington, UK) set at 37°C. Polystyrene oviposition pots (5 cm diameter containing 10 ml distilled H_2_O) were placed into each of the three cages 2 days post blood-meal. Hatched larvae from crosses set up in this way were used in the experiments detailed below.

### Larval Survival, Age at Pupation and Sex Ratio

Exactly 30 L_1_ stage larvae were counted into each of 15 tubs (18.5 cm×10 cm×4.5 cm high) with 150 ml of distilled water containing Liquifry (Interpret Ltd., Dorking, U.K.). Tubs were fed daily on ground TetraMin fish flake (Melle, Germany) using a scaled down version of the standardised protocol below to ensure uniformity and water levels were kept constant throughout the experiment by addition of ddH_2_O when necessary. Larvae were counted daily and dead larvae and pupae were removed. The number of male and female pupae per tub for each strain was recorded daily. Three independent replicates, derived from independently reared adults were undertaken.

### Adult Longevity

Experimental mosquitoes were generated using a standardised rearing protocol which ensured that both wild-type and hemizygous strain adults would be comparable. For wild-type KIL and both reciprocal hemizygous transgenic strains, L_1_ stage larvae were counted into trays at the standard density of 0.2 larvae per ml and distilled water containing 4 drops per litre Liquifry (Interpret Ltd., Dorking, U.K.). Thereafter a strict larval feeding regime was applied to all trays, using ground TetraMin fish flake or TetraMin baby (Melle, Germany) measured in a stainless steel microspoon, supplemented with Tetra Pond Sticks (Melle, Germany) at weekends. Trays of 200 larvae were fed ½ flat spoon on day 2, 1 flat spoon on day 4 and 2 flat spoons plus 12 pond sticks on day 5. Water was topped up to the original level on day 5. From day 7 onwards trays were given 1 flat spoon twice a day until all had pupated. Larval trays were rotated daily within the insectary to encourage synchronous growth and pupation. For initial longevity studies the resulting pupae were picked and sexed daily. Once emerged, groups of ten adults of the same age and sex were placed in clear plastic pots (10 cm height x 11 cm diameter) with 10 replicates of each sex per strain. For longevity studies on mated females, pupae of both sexes were collected and newly eclosed adults collected daily. Two days post-eclosion mosquitoes were sugar starved overnight. The following day, females of each strain were placed into eight separate 15 cm^3^ cages, 25 in 4 cages for non-fed samples and 30 in the remaining 4 cages for blood-feeding. Following the blood-meal any non-fed, partially-fed or excess females were removed to leave 25 in all cages. Fed cages were offered oviposition sites 2 and 3 days post-blood-meal and all cages were sugar starved for 3 hrs prior to offering a second blood-meal 8 days post-eclosion. Pots and cages were placed in the insectary in such a way as to minimise placement effects and rotated daily. Adults were maintained on water and sugar cubes available *ad libitum*. Dead mosquitoes were removed daily and counted for each population within each strain. The experiment ended when all mosquitoes in all populations were dead. Three independent replicates were undertaken using independently reared adults for both experiments.

### Wing Lengths

Adult mosquitoes from the wild-type strain and both reciprocal hemizygous strains were removed daily from pots and cages involved in the longevity studies and stored in 70% ethanol. These mosquitoes, reared following a standardised protocol (details above) had wing lengths measured as an indicator of body size. One wing was removed from each mosquito and measured from the distal end of the allula to the tip, excluding the fringe, using a dissecting microscope and an eye piece graticule calibrated using a 1 mm stage micrometer ruler. Only intact wings were measured.

### Fecundity and Fertility

Excess females reared for the second longevity study, which had been blood-fed 3 days post-eclosion, were used for fecundity studies. Fully engorged females were maintained on water soaked cotton wool and sugar cubes. At 3 days post-blood-meal single females were placed into 75 mm×25 mm (diameter), capped, soda lime glass specimen tubes (Fisher Scientific, Loughborough, UK), lined to 3 cm with filter paper and filled to 1.5 cm with distilled water. Tubes were examined daily. Once egg batches had been laid females were removed. Post-hatching, filter papers were slid out of the tube to collect eggs and stored in Petri dishes for counting. Total eggs laid per female (including any not collected on filter paper) were counted using a dissecting microscope, together with the number from each batch that had hatched.

## References

[1] Ranson H, Abdallah H, Badolo A, Guelbeogo WM, Kerah-Hinzoumbe C, et al (2009) Insecticide resistance in *Anopheles gambiae*: data from the first year of a multi-country study highlight the extent of the problem. Malar J 8: 299. 20015411 10.1186/1475-2875-8-299 PMC2804687

[2] Ito J, Ghosh A, Moreira LA, Wimmer EA, Jacobs-Lorena M (2002) Transgenic anopheline mosquitoes impaired in transmission of a malaria parasite. Nature 417: 452–455. 12024215 10.1038/417452a

[3] Moreira LA, Ito J, Ghosh A, Devenport M, Zieler H, et al (2002) Bee venom phospholipase inhibits malaria parasite development in transgenic mosquitoes. J Biol Chem 277: 40839–40843. 12167627 10.1074/jbc.M206647200

[4] Abraham EG, Pinto SB, Ghosh A, Vanlandingham DL, Budd A, et al (2005) An immune-responsive serpin, SRPN6, mediates mosquito defense against malaria parasites. Proc Natl Acad Sci U S A 102: 16327–16332. 16260729 10.1073/pnas.0508335102 PMC1283470

[5] Yoshida S, Shimada Y, Kondoh D, Kouzuma Y, Ghosh AK, et al (2007) Hemolytic C-type lectin CEL-III from sea cucumber expressed in transgenic mosquitoes impairs malaria parasite development. PLoS Pathog 3: e192. 18159942 10.1371/journal.ppat.0030192 PMC2151087

[6] Corby-Harris V, Drexler A, Watkins de Jong L, Antonova Y, Pakpour N, et al (2010) Activation of Akt signaling reduces the prevalence and intensity of malaria parasite infection and lifespan in *Anopheles stephensi* mosquitoes. PLoS Pathog 6: e1001003. 20664791 10.1371/journal.ppat.1001003 PMC2904800

[7] Rodrigues FG, Santos MN, de Carvalho TX, Rocha BC, Riehle MA, et al (2008) Expression of a mutated phospholipase A2 in transgenic *Aedes fluviatilis* mosquitoes impacts *Plasmodium gallinaceum* development. Insect Mol Biol 17: 175–183. 18353106 10.1111/j.1365-2583.2008.00791.x PMC4137777

[8] Fu G, Lees RS, Nimmo D, Aw D, Jin L, et al (2010) Female-specific flightless phenotype for mosquito control. Proc Natl Acad Sci U S A 107: 4550–4554. 20176967 10.1073/pnas.1000251107 PMC2826341

[9] Meredith JM, Basu S, Nimmo DD, Larget-Thiery I, Warr EL, et al (2011) Site-specific integration and expression of an anti-malarial gene in transgenic *Anopheles gambiae* significantly reduces *Plasmodium* infections. PLoS One 6: e14587. 21283619 10.1371/journal.pone.0014587 PMC3026776

[10] Isaacs AT, Jasinskiene N, Tretiakov M, Thiery I, Zettor A, et al (2012) Transgenic *Anopheles stephensi* coexpressing single-chain antibodies resist *Plasmodium falciparum* development. Proc Natl Acad Sci U S A 109: E1922–1930. 22689959 10.1073/pnas.1207738109 PMC3396534

[11] Isaacs AT, Li F, Jasinskiene N, Chen X, Nirmala X, et al (2011) Engineered resistance to *Plasmodium falciparum* development in transgenic *Anopheles stephensi* . PLoS Pathog 7: e1002017. 21533066 10.1371/journal.ppat.1002017 PMC3080844

[12] Li C, Marrelli MT, Yan G, Jacobs-Lorena M (2008) Fitness of transgenic *Anopheles stephensi* mosquitoes expressing the SM1 peptide under the control of a vitellogenin promoter. J Hered 99: 275–282. 18334506 10.1093/jhered/esn004 PMC4154370

[13] Koenraadt CJ, Kormaksson M, Harrington LC (2010) Effects of inbreeding and genetic modification on *Aedes aegypti* larval competition and adult energy reserves. Parasit Vectors 3: 92. 20925917 10.1186/1756-3305-3-92 PMC2967506

[14] Marrelli MT, Moreira CK, Kelly D, Alphey L, Jacobs-Lorena M (2006) Mosquito transgenesis: what is the fitness cost? Trends Parasitol 22: 197–202. 16564223 10.1016/j.pt.2006.03.004

[15] Moreira LA, Wang J, Collins FH, Jacobs-Lorena M (2004) Fitness of anopheline mosquitoes expressing transgenes that inhibit *Plasmodium* development. Genetics 166: 1337–1341. 15082552 10.1534/genetics.166.3.1337 PMC1470781

[16] Nimmo DD, Alphey L, Meredith JM, Eggleston P (2006) High efficiency site-specific genetic engineering of the mosquito genome. Insect Mol Biol 15: 129–136. 16640723 10.1111/j.1365-2583.2006.00615.x PMC1602059

[17] Grossman GL, Rafferty CS, Clayton JR, Stevens TK, Mukabayire O, et al (2001) Germline transformation of the malaria vector, *Anopheles gambiae*, with the *piggyBac* transposable element. Insect Mol Biol 10: 597–604. 11903629 10.1046/j.0962-1075.2001.00299.x

[18] Catteruccia F, Godfray HC, Crisanti A (2003) Impact of genetic manipulation on the fitness of *Anopheles stephensi* mosquitoes. Science 299: 1225–1227. 12595691 10.1126/science.1081453

[19] O’Brochta DA, Sethuraman N, Wilson R, Hice RH, Pinkerton AC, et al (2003) Gene vector and transposable element behaviour in mosquitoes. J Exp Biol 206: 3823–3834. 14506218 10.1242/jeb.00638

[20] Bellen HJ, Levis RW, He Y, Carlson JW, Evans-Holm M, et al (2011) The *Drosophila* gene disruption project: progress using transposons with distinctive site specificities. Genetics 188: 731–743. 21515576 10.1534/genetics.111.126995 PMC3176542

[21] Meredith JM, Underhill A, McArthur CC, Eggleston P (2013) Next-Generation Site-Directed Transgenesis in the Malaria Vector Mosquito *Anopheles gambiae*: Self-Docking Strains Expressing Germline-Specific phiC31 Integrase. PLoS One 8: e59264. 23516619 10.1371/journal.pone.0059264 PMC3596282

[22] Marrelli MT, Li C, Rasgon JL, Jacobs-Lorena M (2007) Transgenic malaria-resistant mosquitoes have a fitness advantage when feeding on *Plasmodium*-infected blood. Proc Natl Acad Sci U S A 104: 5580–5583. 17372227 10.1073/pnas.0609809104 PMC1838510

[23] Amenya DA, Bonizzoni M, Isaacs AT, Jasinskiene N, Chen H, et al (2010) Comparative fitness assessment of *Anopheles stephensi* transgenic lines receptive to site-specific integration. Insect Mol Biol 19: 263–269. 20113372 10.1111/j.1365-2583.2009.00986.x PMC2862888

[24] Dong Y, Das S, Cirimotich C, Souza-Neto JA, McLean KJ, et al (2011) Engineered *anopheles* immunity to *Plasmodium* infection. PLoS Pathog 7: e1002458. 22216006 10.1371/journal.ppat.1002458 PMC3245315

[25] Bargielowski I, Nimmo D, Alphey L, Koella JC (2011) Comparison of life history characteristics of the genetically modified OX513A line and a wild type strain of *Aedes aegypti* . PLoS One 6: e20699. 21698096 10.1371/journal.pone.0020699 PMC3117796

[26] Irvin N, Hoddle MS, O’Brochta DA, Carey B, Atkinson PW (2004) Assessing fitness costs for transgenic *Aedes aegypti* expressing the GFP marker and transposase genes. Proc Natl Acad Sci U S A 101: 891–896. 14711992 10.1073/pnas.0305511101 PMC321777

[27] Arrighi RB, Nakamura C, Miyake J, Hurd H, Burgess JG (2002) Design and activity of antimicrobial peptides against sporogonic-stage parasites causing murine malarias. Antimicrob Agents Chemother 46: 2104–2110. 12069961 10.1128/AAC.46.7.2104-2110.2002 PMC127320

[28] Groth AC, Fish M, Nusse R, Calos MP (2004) Construction of transgenic *Drosophila* by using the site-specific integrase from phage phiC31. Genetics 166: 1775–1782. 15126397 10.1534/genetics.166.4.1775 PMC1470814

[29] Diaz H, Ramirez AA, Olarte A, Clavijo C (2011) A model for the control of malaria using genetically modified vectors. J Theor Biol 276: 57–66. 21300074 10.1016/j.jtbi.2011.01.053

[30] Paaijmans KP, Wandago MO, Githeko AK, Takken W (2007) Unexpected high losses of *Anopheles gambiae* larvae due to rainfall. PLoS One 2: e1146. 17987125 10.1371/journal.pone.0001146 PMC2063461

[31] Kirby MJ, Lindsay SW (2009) Effect of temperature and inter-specific competition on the development and survival of *Anopheles gambiae sensu stricto* and *An. arabiensis* larvae. Acta Trop 109: 118–123. 19013420 10.1016/j.actatropica.2008.09.025

[32] Edwards MJ, Lemos FJ, Donnelly-Doman M, Jacobs-Lorena M (1997) Rapid induction by a blood meal of a carboxypeptidase gene in the gut of the mosquito *Anopheles gambiae* . Insect Biochem Mol Biol 27: 1063–1072. 9569647 10.1016/s0965-1748(97)00093-3

[33] Diabate A, Yaro AS, Dao A, Diallo M, Huestis DL, et al (2011) Spatial distribution and male mating success of *Anopheles gambiae* swarms. BMC Evol Biol 11: 184. 21711542 10.1186/1471-2148-11-184 PMC3146442

[34] Koella JC, Lyimo EO (1996) Variability in the relationship between weight and wing length of *Anopheles gambiae* (Diptera: Culicidae). J Med Entomol 33: 261–264. 8742532 10.1093/jmedent/33.2.261

[35] Lyimo EO, Takken W (1993) Effects of adult body size on fecundity and the pre-gravid rate of *Anopheles gambiae* females in Tanzania. Med Vet Entomol 7: 328–332. 8268486 10.1111/j.1365-2915.1993.tb00700.x

[36] Hogg JC, Thomson MC, Hurd H (1996) Comparative fecundity and associated factors for two sibling species of the *Anopheles gambiae* complex occurring sympatrically in The Gambia. Med Vet Entomol 10: 385–391. 8994142 10.1111/j.1365-2915.1996.tb00761.x

[37] Okanda FM, Dao A, Njiru BN, Arija J, Akelo HA, et al (2002) Behavioural determinants of gene flow in malaria vector populations: *Anopheles gambiae* males select large females as mates. Malar J 1: 10. 12296972 10.1186/1475-2875-1-10 PMC140138

[38] Nghabi KR, Huho BJ, Nkwengulila G, Killeen GF, Knols BGJ, et al (2008) Sexual selection in mosquito swarms: may the best man lose? Animal Behaviour 76: 105–112.

[39] Dao A, Kassogue Y, Adamou A, Diallo M, Yaro AS, et al (2010) Reproduction-longevity trade-off in *Anopheles gambiae* (Diptera: Culicidae). J Med Entomol 47: 769–777. 20939369 10.1603/me10052 PMC2965199

[40] Briegel H (1990) Fecundity, metabolism, and body size in *Anopheles* (Diptera: Culicidae), vectors of malaria. J Med Entomol 27: 839–850. 2231621 10.1093/jmedent/27.5.839

[41] Huho BJ, Ng’habi KR, Killeen GF, Nkwengulila G, Knols BG, et al (2007) Nature beats nurture: a case study of the physiological fitness of free-living and laboratory-reared male *Anopheles gambiae* s.l. J Exp Biol 210: 2939–2947. 17690243 10.1242/jeb.005033

[42] An C, Budd A, Kanost MR, Michel K (2011) Characterization of a regulatory unit that controls melanization and affects longevity of mosquitoes. Cell Mol Life Sci 68: 1929–1939. 20953892 10.1007/s00018-010-0543-z PMC3070200

[43] Lambrechts L, Morlais I, Awono-Ambene PH, Cohuet A, Simard F, et al (2007) Effect of infection by *Plasmodium falciparum* on the melanization immune response of *Anopheles gambiae* . Am J Trop Med Hyg 76: 475–480. 17360870

